# Endoscopic ultrasonic resection of calcified tumor of the third ventricle

**DOI:** 10.3171/2023.1.FOCVID22143

**Published:** 2023-04-01

**Authors:** Pietro Spennato, Nicola Onorini, Francesca Vitulli, Alessia Imperato, Lucia De Martino, Claudio Ruggiero, Giuseppe Cinalli

**Affiliations:** 1 Department of Pediatric Neurosurgery, Santobono-Pausilipon Children’s Hospital AORN, Naples;; 2 Division of Neurosurgery, Department of Neurosciences, Reproductive and Odonotostomatological Sciences, Università degli Studi di Napoli "Federico II," Naples; and; 3 Department of Pediatric Neurooncology, Santobono-Pausilipon Children’s Hospital AORN, Naples, Italy

**Keywords:** low-grade tumor, child, third ventricle, neuroendoscopy, cavitron

## Abstract

In this video, the authors present ultrasonic resection of calcified tumor of the third ventricle in a 12-year-old boy. He presented to the emergency department with a 1-week history of headache, drowsiness, and bilateral papilledema. Despite extensive calcification visible on a CT scan, a minimally invasive pure endoscopic approach was chosen. The use of an ultrasonic aspirator allows fast and safe removal of the tumor. The histological diagnosis was a low-grade glioneuronal tumor. In conclusion, the endoscopic ultrasonic aspirator is a useful tool to resect tumors in the ventricular system. The presence of calcifications within the tumor does not contraindicate an endoscopic approach.

The video can be found here: https://stream.cadmore.media/r10.3171/2023.1.FOCVID22143

## Transcript

### 0:24 Clinical Presentation.

In this video, we present endoscopic ultrasonic resection of calcified tumor of the third ventricle in a 12-year-old boy.

### 0:30 Radiology.

Radiology showed tumor inside the third ventricle blocking both foramina of Monro with hydrocephalus. Tumor was bulging into a right foramen of Monro and occupying completely the third ventricle with a calcified core, well visible at CT scan.

### 0:44 Equipment.

This is the material that was used for the surgery.^1–6^

### 0:48 Planning of the Trajectory.

This is the trajectory that was chosen through the right frontal horn where the tumor was bulging through the left foramen of Monro.^2,5^ And this is the final trajectory to the target. Right coronal skin incision was performed, planned to be included in a bicoronal incision.

### 1:05 Endoscopic Vision.

This is the endoscopic vision: tumor is well visible bulging inside the foramen of Monro. The foramen of Monro is enlarged by the tumor.

### 1:19 Introduction of the Ultrasonic Aspirator and Initial Debulking.

This is the introduction of the ultrasonic aspirator cannula that is approached to the tumor. And we start to test the texture of the tumor to see if it is feasible a resection or extended resection with the endoscopic ultrasonic aspirator. You see that the tumor is not bleeding very much. Here you can see the accidental aspiration of the choroid plexus, that is better to coagulate before performing any kind of surgery at the level of the foramina of Monro. But you see that the bleeding is very, very limited, and this part of the tumor that is bulging inside the lateral ventricle is certainly not very hemorrhagic and very easy to remove in this way. The cavitron is used with the power that is not exceeding the 50% or 60% of the sonication power, and the aspiration is usually left between 20% and 30%. We don’t want excessive aspiration of fluid in order to avoid sudden decrease in intraventricular pressure. You can see that keeping the cannula just on the surface of the tumor without entering inside the tumor with the cannula, we can remain very safe also on the boundaries: the tumor is attracted inside the cannula and is occupying the aspirating surface of the cannula, so only the tumor is aspirated inside the cannula, and is fragmented by the sonication inside the cannula lumen, and we don’t take any risk.

### 3:02 Tumor Debulking.

Here we are approaching the pillar on the fornix. The bleeding starts to become a little bit more important, but the large ventricular chamber helps us to keep the vision extremely acceptable throughout the procedure.

### 3:19 Dissection From the Fornix.

And here we are removing the adhesion of the tumor at the level of the pillar of the fornix and very close to the choroid plexus and very close to the anterior septal vein.

### 3:30 Approaching the Calcified Core.

Here we are approaching the central calcified core. Central calcified core, we knew from the previous CT scan data, would have been the most difficult part because of the texture and because it is inside the lumen of the third ventricle. You can see very nicely the calcification, and we have to work much closer to the tumor in this area because of the more important bleeding that we can observe inside the calcified core. Working very, very close to the tumor, we can create a microchamber of continuous irrigation that keeps clean the fluid inside the microchamber of work ahead of the endoscope. We can keep under very, very tight visual control the tip of our ultrasonic aspirator that you see is only remaining on the surface of the tumor and just removing and debulking progressively the tumor very slowly, and the calcified areas of the tumor are well visible. Fortunately, they are scattered throughout the tumor, so it is possible to create small fragments of tumor that can be aspirated separately, and although it is certainly more long than complicated than friable part that was inside the ventricular lumen, here we can create anyway a very significant debulking of the central part of the tumor.

### 5:11 Further Debulking.

And then we arrived to the deepest part of the tumor, where the calcified core is finished. We find again another texture of tumor inside the lower part of the third ventricle. Color of the tumor and the consistency of the tumor is well recognizable if compared to the normal ependyma.

### 5:35 Biopsy and Debulking From the Third Ventricle.

At the end of the surgery, we remove some larger piece of tumor together with some clots that are visible inside the tumor, and now we are approaching the boundaries of the tumor. Both the boundaries that are below the pillar of the fornix, some remnant of the calcified part that is still remaining floating inside the third ventricle. Significant irrigation is necessary, in order to have a good vision and avoid deepening the cannula inside healthy normal tissue, of course.

### 6:15 Dissection and Debulking of the Anterior Part.

And here you can see that some part is still attached to the anterior pillar of the fornix, we can do still some job, but the pillar of the fornix is visible and it looks quite healthy, so we can just clean the boundaries of the pillar of the fornix, very nicely respecting the ependyma and respecting certainly the anterior pillar of the fornix that should not be injured by our ultrasonic aspirator.

### 6:45 Final Debulking and Third Ventriculostomy.

Here you can see the normal ependyma on the floor of the third ventricle. The tumor fortunately is easy to recognize and to detach from the normal ependyma. And here you can see the tuber cinereum below the tumor. And after some other irrigation, we can see the floor of the third ventricle. We perform endoscopic third ventriculostomy systematically to facilitate CSF circulation at the level of the third ventricle.

### 7:19 Postoperative Course and Imaging.

Surgical operative time is 115 minutes. These are the details. This is the postoperative MRI showing a subtotal removal with just some very small remnants adherent and below the tela choroidea, and even the calcified core of the tumor has been completely removed. Here you can see that the third ventricle is completely free of tumor, and it is the MRI also showing very nice removal.

### 7:49 Conclusions.

In conclusion, endoscopic ultrasonic aspirator is a useful tool to resect tumor in the ventricular system.^1–6^ Presence of calcifications within the tumor does not contraindicate endoscopic approach.

## Disclosures

The authors report no conflict of interest concerning the materials or methods used in this study or the findings specified in this publication.

## Author Contributions

Primary surgeon: Cinalli. Assistant surgeon: Spennato, Ruggiero. Editing and drafting the video and abstract: Spennato, Onorini, Vitulli, Imperato, De Martino, Cinalli. Critically revising the work: Spennato, Onorini, Imperato, De Martino, Cinalli. Reviewed submitted version of the work: Spennato, Onorini, Vitulli. Approved the final version of the work on behalf of all authors: Spennato. Supervision: Spennato, Cinalli.
